# Increased NK Cell Maturation in Patients with Acute Myeloid Leukemia

**DOI:** 10.3389/fimmu.2015.00564

**Published:** 2015-11-06

**Authors:** Anne-Sophie Chretien, Samuel Granjeaud, Françoise Gondois-Rey, Samia Harbi, Florence Orlanducci, Didier Blaise, Norbert Vey, Christine Arnoulet, Cyril Fauriat, Daniel Olive

**Affiliations:** ^1^Centre de Cancérologie de Marseille, Team Immunity and Cancer, INSERM, U1068, Institut Paoli-Calmettes, Aix-Marseille Université, UM 105, CNRS, UMR7258, Marseille, France; ^2^Centre de Cancérologie de Marseille, Systems Biology Platform, INSERM, U1068, Institut Paoli-Calmettes, Aix-Marseille Université, UM 105, CNRS, UMR7258, Marseille, France; ^3^Centre de Cancérologie de Marseille, Plateforme d’Immunomonitoring en Cancérologie, INSERM, U1068, Institut Paoli-Calmettes, Aix-Marseille Université, UM 105, CNRS, UMR7258, Marseille, France; ^4^Hematology and Transplant and Cellular Therapy Department, Institut Paoli-Calmettes, Marseille, France; ^5^Hematology Department, Institut Paoli-Calmettes, Marseille, France; ^6^Biopathology Department, Institut Paoli Calmettes, Marseille, France

**Keywords:** AML, NK maturation, automated gating, FLOCK algorithm, multidimensional flow cytometry

## Abstract

Understanding immune alterations in cancer patients is a major challenge and requires precise phenotypic study of immune subsets. Improvement of knowledge regarding the biology of natural killer (NK) cells and technical advances leads to the generation of high dimensional dataset. High dimensional flow cytometry requires tools adapted to complex dataset analyses. This study presents an example of NK cell maturation analysis in Healthy Volunteers (HV) and patients with Acute Myeloid Leukemia (AML) with an automated procedure using the FLOCK algorithm. This procedure enabled to automatically identify NK cell subsets according to maturation profiles, with 2D mapping of a four-dimensional dataset. Differences were highlighted in AML patients compared to HV, with an overall increase of NK maturation. Among patients, a strong heterogeneity in NK cell maturation defined three distinct profiles. Overall, automatic gating with FLOCK algorithm is a recent procedure, which enables fast and reliable identification of cell populations from high-dimensional cytometry data. Such tools are necessary for immune subset characterization and standardization of data analyses. This tool is adapted to new immune cell subsets discovery, and may lead to a better knowledge of NK cell defects in cancer patients. Overall, 2D mapping of NK maturation profiles enabled fast and reliable identification of NK cell subsets.

## Introduction

Natural Killer (NK) cells are immune effectors that play a key role in tumor rejection, with an ability to detect and lyse tumor cells without prior stimulation (1–3). Their fundamental role in anti-tumor immune response has been widely demonstrated in both solid tumors and malignant hemopathies, and parameters linked to NK cell activation can either be prognostic factors (4–9) or predictive markers of response to chemotherapy or radiotherapy (10, 11). Thus, monitoring NK cell parameters seems to be an important point to stratify patients at diagnosis and to assess NK cell response during the course of treatment. For such applications, NK cell alterations in cancer patients need to be further described in order to dissect mechanisms involved and define the relevant therapeutic strategies based on NK restoration (12, 13).

In addition to classical NK activating receptors, maturation is fundamental for triggering immune response while maintaining self tolerance (14). NK cell maturation and activation are intrinsically linked (14). Therefore, this point is probably of primary importance for NK cell reactivity in the context of malignancies. Recent studies highlight increasing number of markers that define NK cell subsets according to maturation parameters. In mice, some parameters appear as relevant markers to define NK cell clusters according to maturation process, such as CD16 or CD11b, CD27, and Mac-1, which define NK subsets with progressive acquisition of NK cell effector functions (14–17). In Humans, four parameters further define NK cell subsets according to the expression of NKG2A, KIR, CD57, and CD56 (16, 18). NK cells initially differentiate from immature CD56^bright^ to CD56^dull^ phenotype, with different functions, including cytotoxicity, cytokine production, and migratory capacities (14, 19, 20). Subsequently, NK cells lose expression of NKG2A, and sequentially express CD57 and KIR. Accordingly, five states of maturation stages are defined according to expression of these markers.

To date, an increasing number of NK cell activation and maturation markers have been described (17, 21). The improvement of knowledge regarding the biology of NK cells led to an increase of markers required to phenotypically and functionally characterize these cells (21). Technological advances in the field of flow and mass cytometry led to the development of complex panels to study NK cells, with subsequent generation of high dimensional dataset (21). In this context, manual processing of the data presents many limitations. First, generation of gates in two dimensions is time-consuming, and subject to operator subjectivity (22). Hence, gating strategy can impact on results and conclusions when multiple gates are drawn [(22); Gondois-Rey et al. manuscript in preparation]. Second, the high number of results generated is sometimes hard to interpret and summarize, in particular when large cohorts of patients are analyzed. Another problem is the global comprehension of a complex system and interpretation of results when conclusions are drawn parameter by parameter. New tools are then required to address these problems. Recently, algorithms for automatic gating and 2D mapping of high dimensional dataset have been developed, such as Spade (23), viSNE (24), flowClust (22), or FLOCK (25, 26). These algorithms combined to classification methods enable the visualization of multiple parameters and summarize information. These approaches are of particular importance to enable data visualization, in particular in the context of study of complex systems such as immunity. The present study is an example of application of NK maturation profiling in Healthy Volunteers (HV) and patients with Acute Myeloid Leukemia (AML) using automated analysis of flow cytometry data.

## Patients and Methods

### Patients and Healthy Volunteers

Fresh peripheral blood samples were prospectively collected from AML patients (*N* = 18) at diagnosis before induction chemotherapy and from aged-matched healthy volunteers (*N* = 18). All participants gave written informed consent in accordance with the Declaration of Helsinki. Patients above 65 years at diagnosis were excluded. The entire research procedure was approved by the ethical review board (Institut Paoli-Calmettes Marseille, France). Table 1 lists the baseline characteristics of patients.

**Table 1:** **Patients characteristics**.

Characteristic		All	AML group 1	AML group 2	AML group 3
Patients (no.)	*N*	18	7	5	6
Age at diagnosis	Mean (SD)	52.2 (13.2)	52.6 (14.8)	56.2 (7.1)	48.4 (16.0)
Sex ratio, M/F		1.57	0.43	4.00	2.00
FAB category	*N* (%)				
M0		2 (11.1)	1 (14.3)	1 (20.0)	0 (0.0)
M1		5 (27.8)	1 (14.3)	2 (40.0)	2 (33.3)
M2		5 (27.8)	2 (28.6)	1 (20.0)	2 (33.3)
M3		0 (0.0)	0 (0.0)	0 (0.0)	0 (0.0)
M4		1 (5.6)	1 (14.3)	0 (0.0)	0 (0.0)
M5		3 (16.7)	2 (28.6)	0 (0.0)	1 (16.7)
M6		1 (5.6)	0 (0.0)	1 (20.0)	0 (0.0)
M7		0 (0.0)	0 (0.0)	0 (0.0)	0 (0.0)
Unclassified		1 (5.6)	0 (0.0)	0 (0.0)	1 (16.7)
Status at diagnosis	*N* (%)				
*De novo*		13 (72.2)	6 (85.7)	4 (80.0)	3 (50.0)
t-AML		4 (22.2)	0 (0.0)	1 (20.0)	3 (50.0)
s-AML		1 (5.6)	1 (14.3)	0 (0.0)	0 (0.0)
White blood cell (10^9^ cells/L)	Median (SD)	9.5 (51.4)	24.7 (41.2)	10.2 (14.0)	7.4 (77.6)
Cytogenetic prognosis	*N* (%)				
1		1 (5.6)	1 (14.3)	0 (0.0)	0 (0.0)
2		12 (66.7)	3 (42.9)	4 (80.0)	5 (83.3)
3		5 (27.8)	3 (42.9)	1 (20.0)	1 (16.7)
ELN					
Favorable		3 (16.7)	1 (14.3)	2 (40.0)	0 (0.0)
Intermediate		10 (55.6)	3 (42.9)	2 (40.0)	5 (83.3)
Unfavorable		5 (27.8)	3 (42.9)	1 (20.0)	1 (16.7)
Blasts (blood) at diagnosis	Mean (SD)	42.6 (34.4)	37.6 (27.7)	53.0 (44.2)	39.8 (37.2)
Blasts (BM) at diagnosis	Mean (SD)	63.7 (29.2)	56.9 (24.9)	73.0 (34.8)	63.8 (32.0)

### Flow Cytometry

A FACS Canto II (BD Biosciences, San Jose, CA, USA) was used for flow cytometry. NK cells from whole blood EDTA were immunostained with Krome Orange-conjugated anti-CD45, Phycoerythrin cyanin 7 (PC7)-conjugated anti-CD3, allophycocyanin (APC)-conjugated anti-CD56, fluorescein isothiocyanate (FITC)-conjugated anti-CD158b1b2j, FITC-conjugated anti-CD158a,h (further referred to as KIR), APC-alexafluor 750 (APC AF 750)-conjugated anti-CD159 (NKG2A), pacific blue-conjugated anti-CD57. All the antibodies used in the study were a kind gift of Beckman Coulter, Marseille, France. Red blood cells were lysed with BD FACS Lysing solution (BD Biosciences) before data acquisition.

### Cluster Identification Procedure

FCS files were read, compensated, transformed, and exported using flowCore (R, Bioconductor) (27). FLOCK algorithm was then applied to each exported data file (26). Resulting gated data were imported with R. Centers of populations were extracted, assembled, and exported as a unique tabulated text file using R. MeV permitted heatmap visualization and hierarchical clustering (28). Centers were clustered using euclidean distance. The tree of centers was cut at a threshold that results in clusters with homogeneous mean fluorescence intensity (MFI) profile. Those clear populations were annotated using expert knowledge. The automated gating and cluster identification procedure was described by Gondois-Rey et al. (manuscript in preparation).

### Statistical Analyses

Statistical analyses were performed using GraphPad Prism (GraphPad Software, San Diego, CA, USA). Differences in the distribution of continuous variables between categories were analyzed by either Mann–Whitney test (for comparison of two groups) or Kruskal–Wallis with Dunns’ post test (comparison of three or more groups). Statistical significance was set at *P* < 0.05.

## Results

### Automatic Gating with FLOCK Provides Reliable Results

Multiparametric flow analysis of NK cell subsets has become complex over recent years with the identification of several subsets of NK cells depending on classical activating receptors, inhibitory receptors, and maturation markers. More importantly, it has become evident that rare subsets may be under or overestimated during manual analysis by different investigators and different software. We have, therefore, searched for a more reliable tool to provide unbiased analysis of NK cell subsets in the peripheral blood of AML patients. First, PBMCs from healthy donors (*N* = 18) and AML patients at diagnosis (*N* = 18) were isolated and stained for CD56, CD3, KIR (see PATIENTS AND METHODS), NKG2A, and CD57. Then CD3^−^CD56^+^ live NK cells were initially manually pre-gated, exported, and then analyzed with FLOCK algorithm.

**Figure 1:**
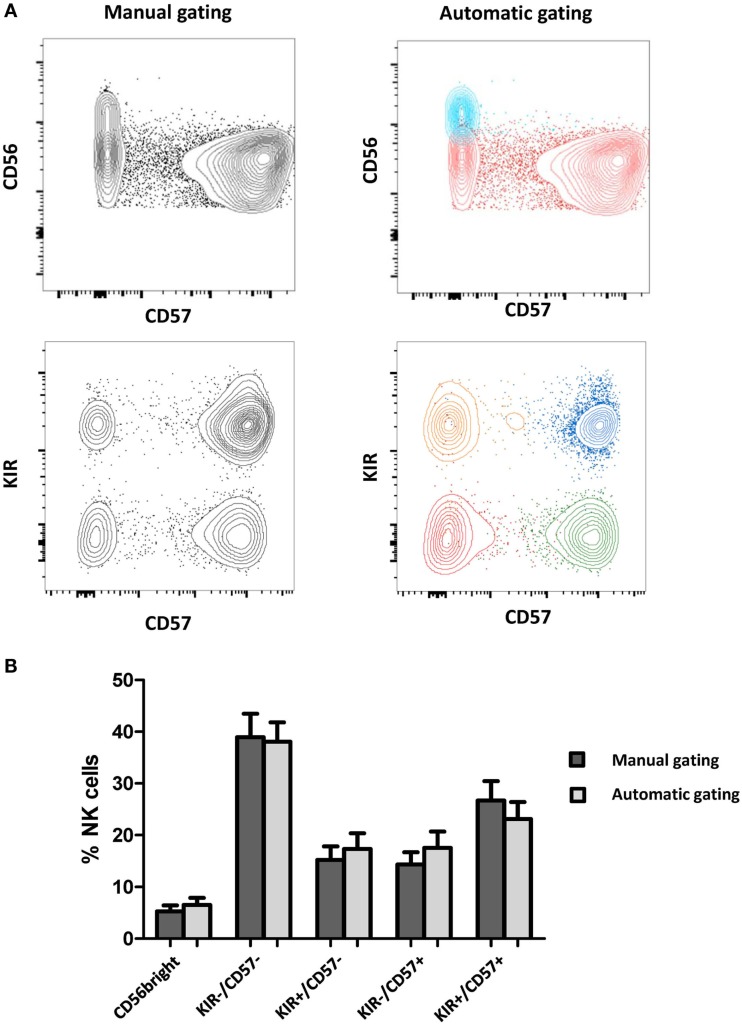
**Comparison of manually gated data and FLOCK analysis**. **(A)** NK clusters were automatically defined by FLOCK and manually annotated as CD56^bright^ or CD56^dim^ NK cells, among which four additional subsets were defined according to KIR and CD57 expression. For a given sample, clusters were defined by FLOCK. The clusters were merged when corresponding to the same NK subpopulation and visualized with FlowJo for comparison with manual gating. Each color represents the merging of clusters corresponding to the same population. **(B)** Frequencies of FLOCK and manually gated subsets of NK cells with respect to CD56, CD57, KIR expression. Data are presented as mean ± SD of Healthy Volunteers (*N* = 18).

Results obtained with manual gating were compared with results obtained with FLOCK. Annotated clusters were merged and graphically compared to the equivalent subsets obtained with manual gating (Figure 1A). Frequencies of FLOCK-gated and manually gated subsets of NK cells in HV samples with respect to CD56, CD57, KIR expression were comparable (Figure 1B). Thus, for each sample, proportions of NK cells within the different clusters with the two approaches were found to be fully consistent.

### Automated Gating with FLOCK Algorithm Evidences NK Subpopulations

**Figure 2:**
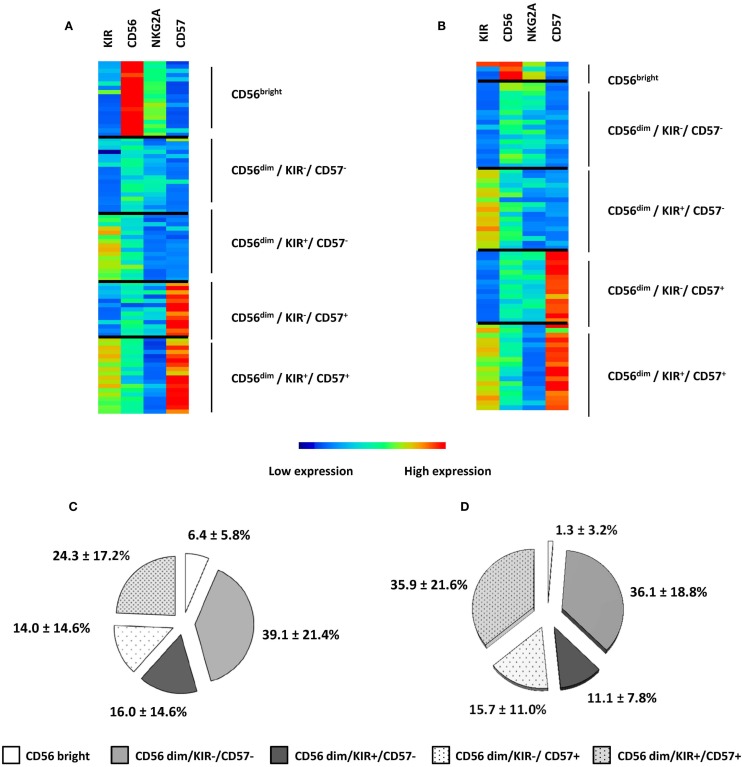
**NK maturation in HV and AML patients**. NK maturation profiles in HV **(A)** and AML patients **(B)** were defined according to FLOCK output and NK subpopulations were represented using Manhattan Hierarchical Clustering based on CD56, KIR, NKG2A, and CD57 expression. Five clusters were defined; each individual is represented in three to five clusters, depending on the presence or absence of NK cells in the different clusters. Percentages of NK cells within clusters are presented as mean ± SD in HV **(C)** and AML **(D)**.

Natural killer cell maturation profiles in HV and AML patients were defined according to FLOCK output. For cluster annotation of FLOCK output data, we used an unsupervised hierarchical clustering with MeV (Figures 2A,B). Overall, the procedure enabled identification of five subsets of NK cells based on the expression of CD56, CD57, KIR, and NKG2A in both patients and healthy volunteers. NK cell differentiate from CD56^bright^ to CD56^dim^ phenotype. CD56^bright^ phenotype then defines the most immature subset of circulating NK cells. In CD56^dim^ NK cells, expression of NKG2A, KIR, and CD57 define several maturation stages (18). Automatic gating procedure with FLOCK enable identification of these different subsets of NK cells, with CD56^bright^ NK cells, and among CD56^dim^ cells, four subsets defined by the positivity or the negativity of KIR and CD57 (Figures 2A,B). In accordance with previous studies, KIR positivity inversely correlates with NKG2A expression, in both HV and AML patients (18, 29). We then considered the repartition of NK cells within the different clusters. On average, the percentage of CD56^bright^ cells was found to be significantly lower in AML patients compared to HV (1.3 ± 3.2% vs. 6.4 ± 5.8%, respectively; *P* = 0.001) (Figures 2C,D).

Overall, 2D mapping of NK maturation profiles enabled the visualization of high dimensional dataset as well as fast and reliable identification of NK cell subsets. With this unsupervised automated gating of NK cells with four parameters, the algorithm was able to distinguish all the NK subsets that were previously described in the literature, but did not identify any new population. Notably, NKG2A was not informative in NK cell cluster definition by the algorithm.

### AML Patients Present Distinct Maturation Profiles

**Figure 3:**
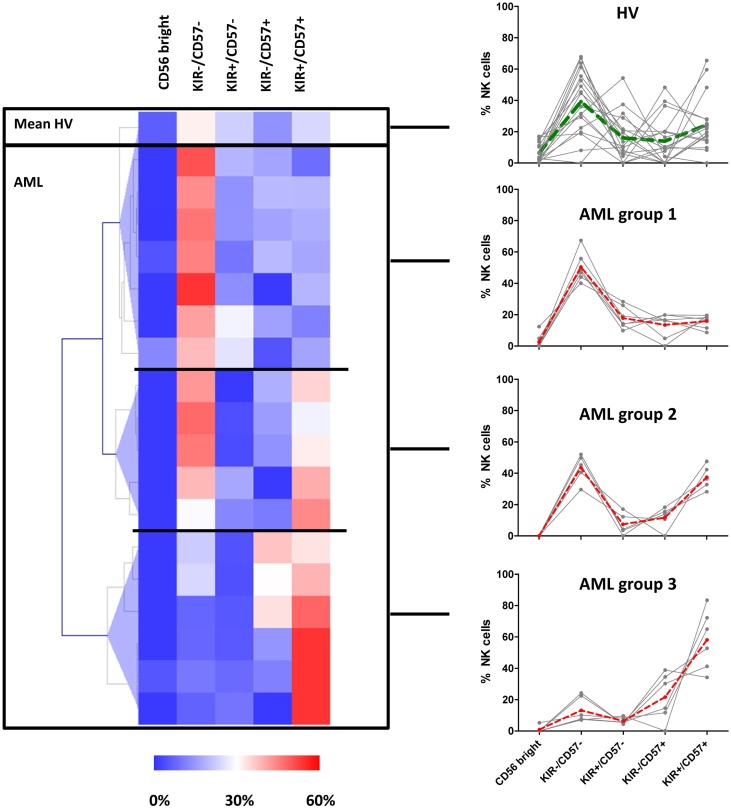
**AML patients can be classified into three distinct groups according to NK maturation profiles**. Left panel: patients and HV were grouped according to the percentage of NK cells represented in the CD56^bright^, KIR^−^/CD57^−^, KIR^+^/CD57^−^, KIR^−^/CD57^+^, KIR^+^/CD57^+^ clusters using hierarchical clustering (HClust, Pearson correlation). This second step of clusterization enabled to define three distinct groups of patients. The frequency of NK cells in each subset for each individual is presented in the right panel. The dashed lines represent the mean frequencies of NK subpopulations in HV and in the three groups of patients.

Patients and HV were clustered according to the percentages of NK cells represented in the CD56^bright^, KIR^−^/CD57^−^, KIR^+^/CD57^−^, KIR^−^/CD57^+^, KIR^+^/CD57^+^ clusters with MeV using unsupervised hierarchical clustering (HClust, Pearson correlation) (Figure 3). This representation allowed defining three distinct groups of patients. The first group of patients (*N* = 7) presented a NK cell maturation profile with 50% (range: 40–67%) NK cells in the cluster KIR^−^/CD57^−^ cluster. Considering the repartition of NK cells within the different clusters, there was no significant difference between this group and HV. The second group of patients (*N* = 5) presented an intermediate maturation profile, with 43% (range: 30–52%) NK cells in the KIR^−^/CD57^−^ cluster and 37% (range: 28–48%) NK cells in the KIR^+^/CD57^+^ cluster. For this group, the proportion of NK cells in the CD56^bright^ cluster was significantly lower than HV (*P* = 0.05). Of note, the apparent high frequency of cells in the cluster KIR^+^/CD57^+^ was not significantly different from HV. The third group of patients (*N* = 6) presented an hyper-maturation profile, with proportions of NK cells of 13% (range: 7–24%) NK cells in the KIR^−^/CD57^−^ cluster and 58% (range: 34–83%) NK cells in the KIR^+^/CD57^+^ cluster. For this group, the proportion of NK cells in the KIR^−^/CD57^−^ cluster was significantly lower than HV (*P* < 0.05) whereas the proportion of NK cells in the KIR^+^/CD57^+^ cluster was significantly higher (*P* < 0.01).

In conclusion, we observed that NK cells in AML patients display marked differences compared to HV, with a strong inter-individual variability, defining three distinct groups of patients according to NK maturation profiles.

## Discussion

Accumulating evidence highlights NK cell parameters as potential prognostic factors in cancer patients, which provides a strong rationale for developing therapeutic strategies aiming at restoring NK cell functions (4–9). However, reaching this point warrants better characterization of NK cell alterations in cancer patients as well as elucidation of the mechanisms involved (30, 31).

Among important parameters involved in NK cell functions, the maturation process is of particular importance; since, depending on the maturation stage NK cells will gain or lose important functions, such as migration capacities, effector functions, response to cytokines, proliferative capacities, IFN-γ production, or cytotoxic activity (8, 14, 32). All these functions are absolutely required for a functional effect against tumor cells. Thus alteration of the maturation process is likely to impact NK cell functions, with direct consequences on patients’ survival (33).

Natural killer cell maturation is a multistep process marked by differential expression of many markers, among which CD56, NKG2A, KIR, and CD57 are of particular importance (18). NK cell subsets can also be defined according to the expression of CD16 and CD56. For instance, it has been described discrete stages of NK cell differentiation. First, CD56^bright^ NK cells expressing low levels of CD16 correspond to a transition between early immature CD56^bright^ CD16^−^ NK cells and CD56^dim^ CD16^+^ NK cells (34). Variations of NK cells in these different compartments have been described in several clinical conditions such as HIV infection and in aging (13). In addition, another NK cell population of CD56^−^CD16^+^ cells has been described and found expanded in particular pathological conditions such as HIV or hepatitis C virus infection (35, 36). Although these discrete stages have been evidenced, the functions of these cells remain elusive, particularly in the context of AML.

Whether NK maturation is impacted by the close proximity with leukemic blasts is an important question. Under physiological conditions, circulating NK cells differentiate from CD56^bright^ to CD56^dim^ phenotype. Then NK cells lose NKG2A expression, and gain KIR expression. CD57 is acquired at later stages of differentiation, and defines a subset of NK cells with low proliferative capacities and high cytotoxic potential (16, 18). In our study, we show that NK cells in AML patients present marked differences compared to Healthy Volunteers. The proportion of CD57^+^ NK cells is increased in one-third of patients, at the expense of less mature NK subsets, with a drastic decrease of immature NK cells. Although CD57^+^ NK cells have been described as the most cytotoxic subset of NK cells, in the context of AML, we still need to check whether these cells display efficient cytotoxic activity on cancer target cells. In addition, the impact of these extreme maturation profiles on clinical outcome warrants further exploration on a larger cohort of patients.

Natural killer cell maturation has been studied in human breast and lung cancer (37, 38). In contrast to our study, tumor-infiltrating NK cells display an immature phenotype, with high percentages of CD56^bright^ NK cells compared to healthy tissues. A notable difference in our study is that we analyzed peripheral blood cells, whereas the previously cited studies were done with infiltrating NK cells. Mamessier et al. hypothesized that NK cells were de-differentiated rather than immature cells; this could explain the high proportion of CD56^bright^ in tumor tissue, without direct impact on central NK maturation or migration of the most immature cells on the tumor site. One additional difference in the context of AML is that NK cells maturate in close contact with tumor cells. This could explain the high proportion of highly maturated NK cells. However, some authors also described CD56^dim^CD57^+^ enrichment in tumor-infiltrated lymph nodes in patients with metastatic melanoma, with significant impact on patients’ survival (33).

Technical advances in flow and mass cytometry now enable the dissection of NK cell biology with high precision, with the subsequent need for tools adapted to the analysis of datasets with unprecedented dimensionality (21, 25, 39). In our study, we used an automated procedure using the FLOCK algorithm and hierarchical clustering, which enabled unsupervised identification of NK subsets and patients profiling based on NK parameters. Automatic gating algorithms are powerful and reliable tools adapted to high dimensional dataset analysis (25, 40) with potential limitations highlighted in the case of rare populations (40, 41). In the case of immunomonitoring studies on large cohorts of patients, the development of such approaches is of primary importance for data analysis standardization. First, the high number of subjects included in these studies requires automated gating in order to reduce the time of analysis. Second, visualization of all the clusters allows fast and unsupervised identification of cell populations. Moreover, the hierarchical classification of patients according to maturation profiles enables the discovery of distinct patterns or specific subgroups among patients. The clinical consequences of such observations should be evaluated on larger cohorts of patients. Considering the potential impact of NK maturation on clinical outcome, NK cell maturation profiling might be informative in prognostic immune signatures and may find applications in patients’ stratification at diagnosis.

## Author Contributions

A-SC: design of the work, data analysis and interpretation, statistical analyses and interface with biological findings, redaction of the article, revisions and final approval of the version to be published, agreement of all aspects of the work in ensuring that questions related to the accuracy or integrity of any part of the work are appropriately investigated and resolved. SG: data interpretation, statistical analyses and interface with biological findings, drafting and revisions of the work, final approval of the version to be published, agreement of all aspects of the work in ensuring that questions related to the accuracy or integrity of any part of the work are appropriately investigated and resolved. FG-R: data interpretation, statistical analyses and interface with biological findings, drafting and revisions of the work, final approval of the version to be published, agreement of all aspects of the work in ensuring that questions related to the accuracy or integrity of any part of the work are appropriately investigated and resolved. SH: data acquisition and interpretation, revisions of the work, final approval of the version to be published, agreement of all aspects of the work in ensuring that questions related to the accuracy or integrity of any part of the work are appropriately investigated and resolved. FO: data analysis and interpretation, drafting of the work and final approval of the version to be published, agreement of all aspects of the work in ensuring that questions related to the accuracy or integrity of any part of the work are appropriately investigated and resolved. DB: conception and design of the work, revisions of the work and final approval of the version to be published, agreement of all aspects of the work in ensuring that questions related to the accuracy or integrity of any part of the work are appropriately investigated and resolved. NV: conception and design of the work, revisions of the work and final approval of the version to be published, agreement of all aspects of the work in ensuring that questions related to the accuracy or integrity of any part of the work are appropriately investigated and resolved. CA: conception and design of the work, revisions of the work and final approval of the version to be published, agreement of all aspects of the work in ensuring that questions related to the accuracy or integrity of any part of the work are appropriately investigated and resolved. CF: conception and design of the work, data analysis and interpretation, article redaction, revisions of the work and final approval of the version to be published, agreement of all aspects of the work in ensuring that questions related to the accuracy or integrity of any part of the work are appropriately investigated and resolved. DO: conception and design of the work, interpretation of data for the work, revisions of the work and final approval of the version to be published, agreement of all aspects of the work in ensuring that questions related to the accuracy or integrity of any part of the work are appropriately investigated and resolved.

## Conflict of Interest Statement

The authors declare that the research was conducted in the absence of any commercial or financial relationships that could be construed as a potential conflict of interest.
